# Cytogenetic and Molecular Characterization of B-Genome Introgression Lines of *Brassica napus* L.

**DOI:** 10.1534/g3.116.036442

**Published:** 2016-11-07

**Authors:** Inderpreet Dhaliwal, Annaliese S. Mason, Shashi Banga, Sakshi Bharti, Beerpal Kaur, Allison Mary Gurung, Phillip Anthony Salisbury, Jacqueline Batley, Surinder Singh Banga

**Affiliations:** * Department of Plant Breeding and Genetics, Punjab Agricultural University, Ludhiana 141001, India; † School of Agriculture and Food Sciences, University of Queensland, Brisbane, Queensland 4072, Australia; ‡ Department of Plant Breeding, Research Centre for Biosystems, Land Use and Nutrition, Justus Liebig University, 35392 Giessen, Germany; § School of Plant Biology, University of Western Australia, Crawley, Western Australia 6009, Australia; ** Institute of Land and Food Resources, University of Melbourne, Parkville 3010, Victoria, Australia

**Keywords:** oilseed rape, siliqua shatter resistance, fl-GISH, graphical genotyping, SNP genotyping

## Abstract

*Brassica napus* introgression lines (ILs), having B-genome segments from *B. carinata*, were assessed genetically for extent of introgression and phenotypically for siliqua shatter resistance. Introgression lines had 7–9% higher DNA content, were meiotically stable, and had almost normal pollen fertility/seed set. Segment introgressions were confirmed by fluorescent genomic *in situ* hybridization (fl-GISH), SSR analyses, and SNP studies. Genotyping with 48 B-genome specific SSRs detected substitutions from B3, B4, B6, and B7 chromosomes on 39 of the 69 ILs whereas SNP genotyping detected a total of 23 B-segments (≥3 Mb) from B4, B6, and B7 introgressed into 10 of the 19 (C1, C2, C3, C5, C6, C8, C9, A3, A9, A10) chromosomes in 17 ILs. The size of substitutions varied from 3.0 Mb on chromosome A9 (IL59) to 42.44 Mb on chromosome C2 (IL54), ranging from 7 to 83% of the recipient chromosome. Average siliqua strength in ILs was observed to be higher than that of *B. napus* parents (2.2–6.0 *vs.* 1.9–4.0 mJ) while siliqua strength in some of the lines was almost equal to that of the donor parent *B. carinata* (6.0 *vs.*7.2 mJ). These ILs, with large chunks of substituted B-genome, can prove to be a useful prebreeding resource for germplasm enhancement in *B. napus*, especially for siliqua shatter resistance.

A relatively young polyploid, *Brassica napus* originated through several hybridization events between its diploid progenitors *B. rapa* (AA; 2*n* = 20) and *B. oleracea* (CC; 2*n* = 18) (Allender and King 2010). The geographic center of origin of *B. napus* is unknown, as records of this crop over the last 500 yr do not predate its origin (Gómez-Campo and Prakash 1999). Although feral populations are common in Europe, there is no evidence of truly wild *B. napus* populations (Prakash *et al.* 2011). Due to its relatively recent origin, probably as an agricultural hybrid, it has low allelic diversity as compared to that of its progenitors. Genetic studies based on DNA polymorphisms show a narrow genetic base (Diers *et al.* 1996; Lombard *et al.* 2000; Cartea *et al.* 2005; Zhou *et al.* 2006; Chen *et al.* 2008; Wang *et al.* 2009; Gyawali *et al.* 2013) with germplasm tending to cluster by growth habit (Qian *et al.* 2006; Zhou *et al.* 2006; Chen *et al.* 2008) or geographic origin (Hu *et al.* 2007). The inherent problem of low genetic diversity in *B. napus* has been further intensified by aggressive plant breeding efforts toward improving oil quality and facilitating adaptation to restricted photoperiod and temperature regimes. Plant breeders are now confronted with diminishing selection gains for yield, oil content, and adaptability to climatic challenges.

Loss of genetic diversity in the modern cultivars of *B. napus* is well documented in Australia (Cowling 2007; Wang *et al.* 2009), Canada (Fu and Gugel 2010), and Germany (Abbadi and Leckband 2011). There is evidence that the C-genome of *B. napus* possesses a relatively narrow genetic base compared to the A-genome (Bus *et al.* 2011). Efforts to broaden genetic diversity in *B. napus* through interspecific hybridization with related species have also benefited the A-genome more than the C-genome (Rahman 2013).

Acquiring new genetic diversity in breeding programs is crucial for continuous germplasm enhancement. Introgression of novel genes from progenitor species through direct resynthesis has shown excellent potential in enhancing heterosis of resynthesized rapeseed genotypes (Girke *et al.* 1999; Gehringer *et al.* 2007). However, a serious limitation to this approach is the association of undesirable linkages in terms of poor seed quality traits and unacceptable agronomic characteristics (Girke *et al.* 1999). In spite of the pervasive limitation of linkage drag, the introgression of exotic alleles from progenitor diploids, related allopolyploids, and wild crucifers is crucial for a continuous pipeline of canola germplasm improvement, both for qualitative and quantitative traits such as pest resistance and productivity.

Development of genetically more diverse *B. napus* lines through substitution of the *B. napus* A-genome with the A-genome of *B. rapa* and substitution of the *B. napus* C-genome with the C-genome of *B. carinata* has also been attempted. This method involved development of trigenomic hexaploids (AABBCC), followed by subsequent backcrossing to natural *B. napus* (Li *et al.* 2004, 2006; Xiao *et al.* 2010). The resulting *B. napus* lines carried >75% of the genome from *B. rapa* (A-genome) and *B. carinata* (C-genome). Introgression lines (ILs) can provide a promising avenue to effectively exploit the genetic potential of related crop allopolyploids and wild species. *B. carinata* (BBCC; 2*n* = 34) is a known source of tolerance to many biotic (Navabi *et al.* 2010) and abiotic stresses. However, attempts to introgress genetic variation from the B-genome of *B. carinata* have largely been unsuccessful; B-genome chromosomes were either eliminated in the early generations (Li *et al.* 2004; Schelfhout *et al.* 2006) or retained as intact (B1, B3, B6, B7 and B8) or broken chromosomes in advanced progenies of interspecific crosses (Navabi *et al.* 2010, 2011; Fredua-Agyeman *et al.* 2014).

In this communication, we report the cytogenetic and molecular characterization of 69 B-genome ILs of *B. napus*. These ILs were synthesized with the aim of introgressing useful gene(s), especially siliqua shatter resistance from B-genome of *B. carinata*, as siliqua shattering is a major commercial cultivation bottleneck in *B. napus*.

## Materials and Methods

### Synthesis of ILs

**Figure 1:**
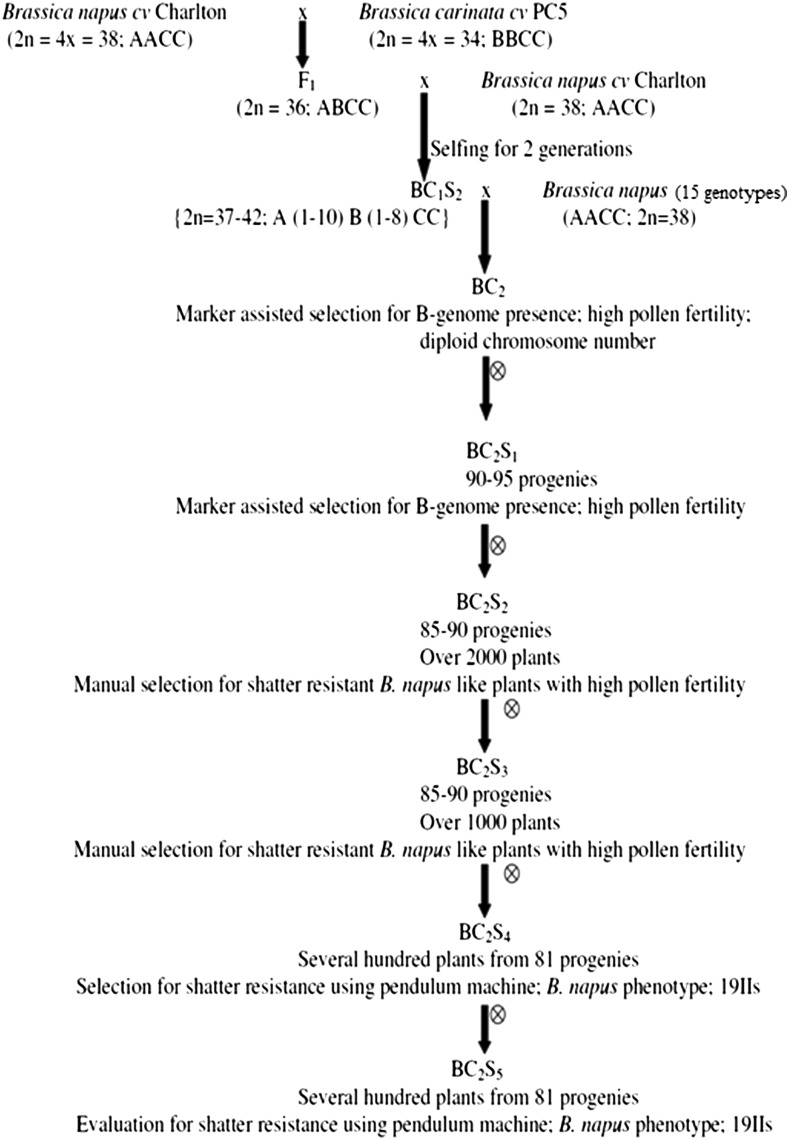
Breeding strategy followed for developing *B. napus–B. carinata* introgression lines. *B. napus* cv. Charlton was crossed with *B. carinata* cv. PC5 and the resultant F_1_ was backcrossed to Charlton. BC_1_ was selfed for two generations to get BC_1_S_2_ which was crossed with 15 diverse *B*. *napus* pollen parents. Resulting 15 BC_2_ progenies were subjected to intense selection and thereafter carried upto BC_2_S_5_ as depicted above.

Continuous attempts have been made over the past 10 years to introgress genes associated with pod shatter resistance from *B. carinata* cv. PC5 into the genetic background of a group of 15 *B. napus* genotypes. The procedure followed for the synthesis of ILs is depicted in Figure 1. The crossing scheme was designed to promote homologous chromosome pairing between the C-genome chromosomes of *B. napus* and *B. carinata*, but homeologous exchanges were also expected to take place between combining B-/A- and B-/C-genomes especially during backcross generations. A large plant population (over 200 plants) was raised in each backcross or selfed generation. Every plant was assayed for pollen grain stainability and also probed using B-genome-specific chromatin marker pBNBH35 (Gupta *et al.* 1992; Schelfhout *et al.* 2004) to identify putative plants with B-genome introgressions. A strong phenotypic selection pressure for siliqua shatter resistance was applied between the BC_2_S_1_ and BC_2_S_5_ generations. Plants with high pollen grain stainability and hard-to-shatter siliquae only were retained for further generations of selfing and backcrossing. A total of 81 ILs were retained at the end of BC_2_S_5._

### Phenotyping of ILs for pod shatter resistance

The set of 81 *B. napus* ILs was raised along with 15 *B. napus* recipient genotypes under two environments during 2012–2013 and one environment during 2013–2014 in an α lattice design with two replications. Paired rows each of *B. carinata* cv. PC5 (donor for siliqua shatter resistance) and *B. nigra* cv. UP (diploid B-genome parent of *B. carinata*) were raised separately, to be used as controls for estimation of siliqua shatter energy as well as for molecular analysis. Since high-quality DNA, required for SNP genotyping, could only be obtained for 69 ILs, five *B. napus* parents, *B. carinata* cv. PC 5, and *B. nigra* cv. UP, this study reports results for molecular analysis on the basis of these lines only.

### Estimation of nuclear DNA content

Self-pollinated progeny from the introgressed material was analyzed in the BC_1_S_5_ generation to determine variation in genome size and ploidy level using a standard flow cytometry DNA content estimation protocol (Doležel and Bartoš 2005). The PartecCyStain UV precise P reagent kit was used for nuclei extraction and DNA staining. Approximately 1–2 mg of young leaf tissue was finely chopped with a sharp razor blade in 400 μl of extraction buffer. The sample was filtered into a test tube containing 1.6 ml staining solution through a 50 μm CellTrics filter. This solution was incubated at room temperature for 30–60 sec and then ∼10,000 nuclei per sample were analyzed using a Partec CyFlow Ploidy Analyzer (Partec, Münster, Germany). Before analyzing samples on a particular day, the flow cytometer was calibrated first using fixed chicken red blood cells, having a known 2C DNA content of 2.33–2.5 pg, and then using an international DNA reference standard *Lycopersicon esculentum* var *stupicke polni tyckoverane*, which has a known 2C DNA content value of 1.96 pg (Doležel *et al.* 1992). *B. carinata* was used as an internal standard, with its DNA content determined in relation to the *Lycopersicon* sample. The absolute DNA amount of a sample was computed using the formula:

$$\left\{\left(\text{sample}\;{\text{G}}_{1}\;\text{peak}\;\text{mean}\right)/\left(\text{standard}\;{\text{G}}_{1}\;\text{peak}\;\text{mean}\right)\right\}\times \text{standard}\;\text{2C}\;\text{DNA}\;\text{content}$$

For determining the genome size, the 1C content of the sample was multiplied by 978 Mb since 1 pg has been reported to be equal to 978 Mb (Doležel *et al.* 2003).

### Cytogenetic studies

Pollen grain stainability was determined from squash preparations of five freshly dehisced anthers in 1% acetocarmine solution. A total of five plants from each genotype were sampled for the purpose. For meiotic studies, squash preparations of anthers at the appropriate stage were made in 2% acetocarmine, and at least 50 PMCs (pollen mother cells) per introgression line with well spread metaphase-I/diakinesis/anaphase-I were observed under an Olympus BX61TRF microscope.

### Fluorescent genomic in situ hybridization (fl-GISH)

The GISH protocol as proposed by Schwarzacher and Heslop-Harrison (2000) was followed with minor modifications, to study B-genome introgressions.

#### Preparation of chromosome spreads:

Flower buds were fixed directly in Farmer’s solution (three parts ethanol: one part acetic acid) in the early morning hours. To remove the fixative, anthers of appropriate size were washed in a citrate buffer for 30 min and subsequently incubated in an enzyme mixture containing 2% (v/w) cellulase and 20% (v/v) pectinase in 4 mmol/l citrate buffer (pH 4.8) for 1 hr at 37°. These anthers were agitated in a microcentrifuge tube to release PMCs, centrifuged for 3 min at 600–800 × *g*, and then treated for 45 min in 150 mmol/l KCl. The PMCs were washed three times at 800 × *g* for 3 min in freshly prepared fixative to clear the cytoplasm. One drop of 7 µl PMC suspension was released on acid-cleaned chilled slides for proper spread. Slides were air-dried in a desiccator before further use.

#### Preparation of probes:

Purified DNA of *B. nigra* cv UP, extracted using the DNeasy plant mini kit (Qiagen), was used for preparing the probe. DNA was sheared in an autoclave for 2 min and then allowed to cool slowly for reannealing. Sheared DNA of the desired fragment size (500–1000 bp) was labeled with Tetramethyl-Rhodamine-5-dUTP (Sigma Aldrich) dye using a nick-translation kit. The DNA of *B. napus* (200–500 bp) was autoclaved and was used as a blocker at 200 times the probe concentration to prevent nonspecific intergenomic cross-hybridization.

#### In situ hybridization:

Forty microliters of hybridization mixture containing 50% formamide, 2× SSC (saline sodium citrate buffer), 10% dextran sulfate, 0.025 µg salmon sperm DNA, 1.25 mM EDTA, 0.1255% acetic acid, 200 ng of labeled probe, and 200-fold blocking DNA was applied on slides with good chromosome spreads, followed by incubation at 80° for 4 min in a thermocycler for simultaneous denaturation of probe and chromosomes. These slides were kept for hybridization overnight at 37° and then washed at 42° for 2 min in 2× SSC and twice for 5 min in 0.1× SSC. Slides were dehydrated through an ethanol series (70, 90, and 96% for 2 min each) and air-dried. Chromosomes were counterstained with DAPI (4′, 6-diamidino-2-phenylindole) by incubating slides with 100 µl DAPI solution (4 µg/ml in McIlvaine’s buffer) at room temperature for 10–30 min in the dark. Slides were rinsed in detection buffer and two drops of antifade was added. Fluorescence was visualized using a Carl Zeiss microscope (Imager Z2AX10). At least 25 cells were photographed using a computer-assisted cooled charge-coupled device camera and images were merged with Image-I software.

### DNA extraction

Fresh leaves of individual ILs were collected at the juvenile plant stage and ground in liquid nitrogen. DNA was extracted from the ground tissues using the CTAB (cetyltrimethylammonium bromide) method (Doyle and Doyle 1990). The DNA was lyophilized and sent to the Centre for Integrative Legume Research (CILR) Laboratory, The University of Queensland, Australia for SNP genotyping.

### B-genome-specific SSR marker allele presence in ILs

The 69 ILs were analyzed using 48 SSR markers (at six markers per chromosome) across the B-genome (Dr. Isobel Parkin, personal communication available under a materials transfer agreement from Agriculture and AgriFood Canada). Known approximate map positions were also supplied for these markers. The 10.3 µl of PCR master mix contained 5 µl of 5 ng/µl template DNA, 1 µl of 10× PCR buffer with 15 mM MgCl_2_ (Sigma Aldrich), 1 µl of 10 µM each primer, 2 µl of 1 mM dNTP_S_ (Sigma Aldrich), and 0.3 µl of 3 U/µl Taq DNA polymerase (Sigma Aldrich). PCR analysis was carried out in 96-well PCR plates using an Eppendorf AG 6325 thermocycler on the following profile: initial cycle of denaturation at 94° for 5 min; 35 amplification cycles each of denaturation at 94° for 30 sec, optimal annealing temperature for 30 sec, elongation at 72° for 1 min, and a final extension cycle at 72° for 7 min. The amplified DNA product was resolved using 6% nondenaturing polyacrylamide gel and visualized using a Syngene Gene Genius gel documentation system. The size of PCR product was recorded with reference to a 100-bp ladder. *B. rapa* (AA), *B. oleracea* (CC), *B. nigra* (BB), *B. carinata* (BBCC), *B. juncea* (AABB), and *B. napus* (AACC) were used as controls. Any marker that showed amplification in *B. rapa* (AA), *B. oleracea* (CC), or *B. napus* (AACC) was rejected. Markers which showed expected size amplification in all three B-genome-bearing species, *i.e.*, *B. nigra* (BB), *B. juncea* (AABB) or *B. carinata* (BBCC), were retained to detect the presence of B-chromosomes in the ILs. Thus, 22 markers representing all the eight B-genome chromosomes amplified on the ILs. Further, introgression was only considered if two or more consecutive markers from a single chromosome were present in an IL. Consequently, 11 markers (SJ7046, SB1752, SB2141AI, SB1935A, SJ1505, SJ0338, SJ0502, SJ7104, SB31138, SJ39119I, SJ13133) representing four chromosomes were such that met these very stringent criteria. Software Graphical GenoTypes v.2.0 (vanBerloo 2008) was used for graphical representation of the polymorphism data thus generated to confirm the presence of B-genome alleles in each IL (Supplemental Material, Figure S1).

### SNP genotyping

An Illumina Infinium 60k SNP array developed for *B. napus* (http://www.illumina.com), carrying 52,157 SNPs across the 19 A- and C-genome chromosomes, was used for genotyping. Hybridization protocols were run as per manufacturer’s specifications for all samples in the population and controls (parent species). The chips were scanned using Illumina HiScanSQ. Genotypic data were visualized using Genome Studio v.2011.1 (Illumina, Inc., San Diego, CA). Single nucleotide polymorphisms were filtered in Genome Studio to retain genome-specic SNPs. Polymorphic SNP markers were distributed evenly across all 19 *B. napus* chromosomes. SNPs that did not show three clear genotype clusters (AA, AB, BB) in the population were removed. As the available SNP chip was designed for A- and C-genomes it could not be used directly to score B-genome chromatin substitutions. All SNPs giving >20% hetero calls (AB), >10% no calls (NC), or cross-amplifying the B-genome of *B. nigra*/*B. carinata* were removed. Finally, 1458 A-genome and 1936 C-genome SNPs (a total of 3394) were retained. NCs were recorded for many SNPs in several regions of ILs. Strings of NCs ≥3 Mb were color coded and treated as B-genome introgressions. All the preexisting A–C translocations in recipient *B. napus* genotypes were not included in the introgression count.

### Phenotyping for resistance to siliqua shatter

The set of 81 ILs and 15 *B. napus* parents grown in the replicated trial under three environments were compared with hard to thresh donor parent *B. carinata* cv. PC5 for introgression of shatter resistance. Five plants were randomly tagged from the two center rows of each plot in each replicate in each environment. Five siliquae from the center of the main raceme were carefully detached at physiological maturity and stored in coarse silica gel blue self-indicating granules to equilibrate them to constant moisture content at room temperature. The siliquae were oven dried at 70° for 24 hr immediately before assessing siliqua strength. The relative resistance to siliqua shatter was measured in terms of rupture energy using an improvised pendulum apparatus (Liu *et al.* 1994; Kadkol 2009) wherein the pendulum strikes the siliqua with a known force and records the energy absorbed to split it open in millijoules.

### Data availability

All data for confirming the conclusions in this research paper are represented in the figures and tables. Detailed data will be made available on request.

## Results

### Genome size variation, meiotic configuration, and pollen fertility in ILs

The 1C genome size in the ILs ranged from 1246 to 1336 Mb (Table 1), which is 7–9% higher than the reported genome size range of 1165–1222 Mb for *B. napus* (http://www.brassica.info/info/reference/genome-sizes.php). Analysis of the meiotic configurations revealed a mode of 19 bivalents at metaphase-I with regular anaphase chromosome separation (Table 1) in all evaluated ILs. Only a few PMCs showed rare univalent (<0.12 per cell) or multivalent (<0.04 per cell) formations. Pollen grain stainability varied from 60% (IL43) to 97.5% (IL11, IL12, IL24, IL46). Seed set on self-pollination was normal, suggesting high female fertility.

**Table 1:** Pollen fertility, meiotic configuration, genome size variation, and B-genome presence in *B. napus* introgression lines

Sr. No.	Introgression Line	1C Genome Size (Mb)	2C DNA Content (pg)	Meiotic Configuration		Pollen Fertility (%)	Presence of B-Genome-Specific Marker “pBNBH35”
				Metaphase	Anaphase		
				(2*n* = 38)	(*n* = 19)		
1	IL1	1280	2.62	─	─	85.0	+
2	IL3	1257	2.57	─	─	92.5	+
3	IL4	─	─	19 IIs	19 Is	97.0	+
4	IL5	1336	2.73	─	─	82.5	+
5	IL6	─	─	─	─	90.0	+
6	IL7	─	─	─	─	90.0	+
7	IL9	1246	2.55	19 IIs	19 Is	92.5	+
8	IL10	1336	2.73	─	─	90.0	+
9	IL11	1308	2.67	─	─	97.5	+
10	IL12	1285	2.63	19 IIs	19 Is	97.5	+
11	IL13	─	─	─	─	87.5	+
12	IL14	1302	2.66	19 IIs	19 Is	90.0	+
13	IL16	1285	2.63	19 IIs	19 Is	90.0	+
14	IL17	─	─	─	─	90.0	+
15	IL18	1274	2.61	─	─	87.5	+
16	IL19	─	─	─	─	90.0	+
17	IL20	1285	2.63	─	─	90.0	−
18	IL21	1274	2.61	─	─	77.5	+
19	IL22	1325	2.71	─	─	97.0	−
20	IL23	1285	2.63	19 IIs	19 Is	82.5	+
21	IL24	1313	2.69	─	─	97.5	+
22	IL26	─	─	─	─	92.5	+
23	IL27	1302	2.66	─	─	85.0	+
24	IL28	─	─	19 IIs	19 Is	67.5	+
25	IL30	─	─	─	─	87.5	−
26	IL31	─	─	─	─	72.5	+
27	IL32	1302	2.66	19 IIs	19 Is	90.0	+
28	IL33	─	─	─	─	77.5	+
29	IL34	1319	2.7	19 IIs	19 Is	87.5	+
30	IL37	─	─	19 IIs	19 Is	67.5	+
31	IL38	1280	2.62	─	─	72.5	+
32	IL39	─	─	19 IIs	19 Is	62.5	+
33	IL40	1268	2.59	19 IIs	19 Is	65.0	+
34	IL41	─	─	19 IIs	19 Is	87.5	+
35	IL42	─	─	19 IIs	19 Is	85.0	+
36	IL43	1280	2.62	─	─	60.0	+
37	IL44	─	─	─	─	75.0	+
38	IL45	1319	2.7	─	─	77.5	+
39	IL46	1330	2.72	─	─	97.5	+
40	IL47	1280	2.62	19 IIs	19 Is	92.5	+
41	IL48	─	─	19 IIs	19 Is	95.0	−
42	IL49	─	─	─	─	85.0	+
43	IL50	1308	2.67	─	─	75.0	+
44	IL51	─	─	─	─	72.5	+
45	IL52	1296	2.65	19 IIs	19 Is	82.5	−
46	IL53	─	─	─	─	77.5	−
47	IL54	─	─	─	─	82.5	+
48	IL56	─	─	─	─	87.5	+
49	IL57	─	─	─	─	62.5	−
50	IL59	─	─	─	─	77.5	+
51	IL61	─	─	─	─	77.5	+
52	IL62	1297	2.65	─	─	90.0	+
53	IL63	─	─	─	─	75.0	−
54	IL64	─	─	─	─	67.5	+
55	IL65	1285	2.63	─	─	77.5	+
56	IL66	1274	2.61	─	─	90.0	−
57	IL67	1274	2.61	─	─	62.5	+
58	IL68	─	─	19 IIs	19 Is	92.5	−
59	IL69	─	─	─	─	87.5	+
60	IL70	1325	2.71	─	─	92.5	+
61	IL71	─	─	─	─	75.0	+
62	IL72	─	─	19 IIs	19 Is	80.0	+
63	IL73	1325	2.71	─	─	90.0	+
64	IL74	─	─	19 IIs	19 Is	60.0	+
65	IL75	─	─	─	─	85.0	+
66	IL76	─	─	─	─	85.0	+
67	IL78	─	─	─	─	85.0	+
68	IL79	1330	2.72	─	─	85.0	+
69	IL80	─	─	19 IIs	19 Is	80.0	+

### Fluorescent genomic in situ hybridization

**Figure 2:**
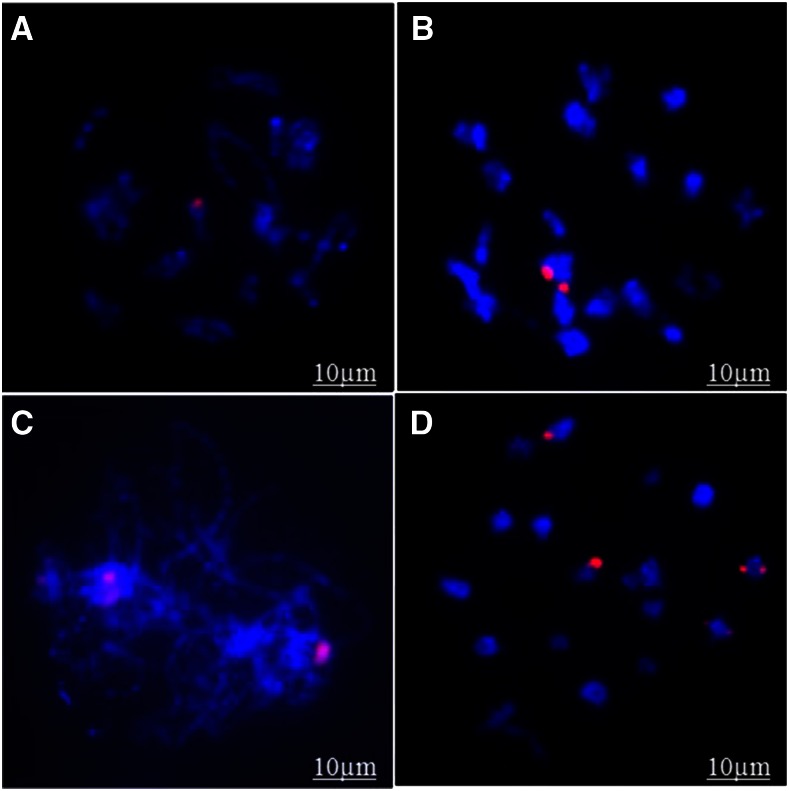
GISH signals showing the substituted B-genome fragment(s) in *B. napus* introgression lines, (A) IL16 at metaphase, (B) IL75 at metaphase, (C) IL69 at pachytene, (D) IL59 at metaphase. All 19 A-/C-genome chromosomes are characterized by blue color due to staining with DAPI. B-genome chromosome introgressions are expressed in red color due to labeling with B-genome specific probe.

GISH with a *B. nigra* chromatin probe did not show any B-genome signal in any of the *B. napus* controls whereas 1–4 bivalents having B-genome fragment substitutions (stained red) were observed in the ILs (Figure 2). The remaining 18-15 A-/C-genome bivalents counter-stained blue with DAPI and had no detectable (≥10 kbp) fragment substitution.

### Genotyping with B-genome-specific SSR primers

**Figure 3:**
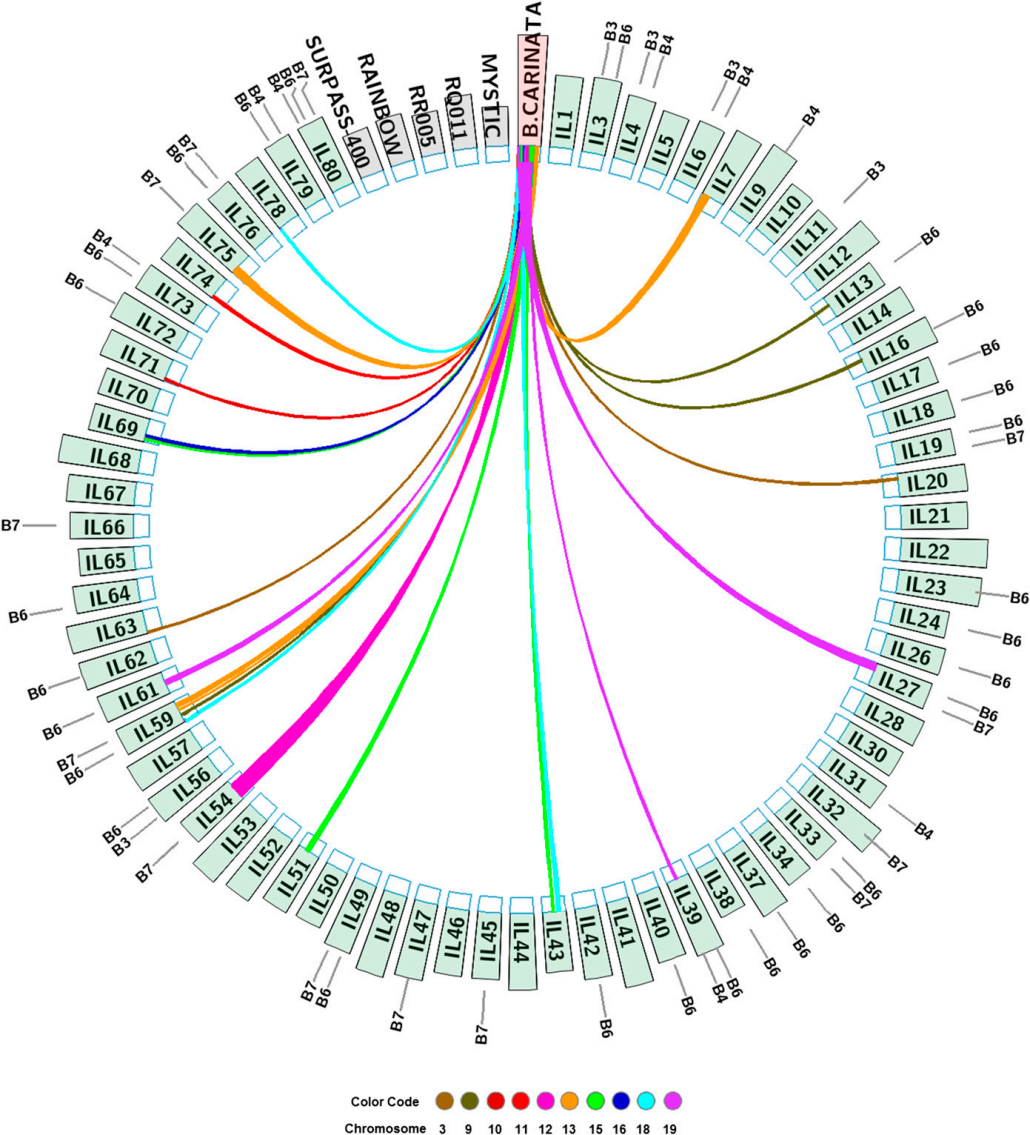
CIRCOS diagram depicting ILs having B-genome fragment introgression(s) > 3 Mb in specific A-/C- chromosomes based on SNP analysis (inner circle), comparative siliqua shatter energy (outer circle) and B-genome chromosomes involved in introgression in different ILs based on SSR analysis (outermost notations).

Molecular genotyping with chromosome-specific SSR markers allowed us to identify B-genome chromosomes involved in the introgressions (Figure 3, Figure S1, and Table 2). Out of the evaluated 48 SSR markers from eight B-genome chromosomes (at six per chromosome), only 22 markers representing all the eight chromosomes got amplified. Further, this number was reduced to only 11 markers (SJ7046, SB1752, SB2141AI, SB1935A, SJ1505, SJ0338, SJ0502, SJ7104, SB31138, SJ39119I, SJ13133) from chromosomes B3, B4, B6, and B7, respectively, due to the stringent criterion followed for marking an introgression as positive, *i.e.*, the amplification of two or more consecutive markers on a single chromosome in an inclusive introgression. On this basis, 39 of the 69 evaluated ILs had introgressed B-genome fragments. Number of fragments introgressed per IL varied from one to five; 13 ILs had 1, 11 had 2, 9 had 3, 5 had 4, and 1 had 5 introgressed fragments. An interstitial marker SJ7046 and a terminal marker SB1752 of chromosome B3 showed amplification in five ILs. Two markers, SB2141AI and SB1935A from chromosome B4 showed amplification in eight ILs (IL4, IL6, IL9, IL31, IL39, IL73, IL79, IL80). Out of the six B6 markers used, five (SB31138, SJ7104, SJ0338, SJ1505, SJ0502) showed amplification in 28 ILs: consecutive markers SB31138 and SJ7104 amplified in 3 ILs (IL27, IL59, IL61); SJ7104 and SJ0338 amplified in 26 ILs and SJ1505 and SJ0502 amplified in 4 ILs. Interestingly all the five polymorphic B6 markers amplified in IL59. Interstitial markers SJ39119I and SJ13133 confirmed the presence of substituted B-genome fragments from chromosome B7 in 14 ILs.

**Table 2:** B-genome chromosome segment substitutions as per SNP assays in the A- and C-genome chromosomes of *B. napus* introgression lines

Introgression Line	Number of Introgressed B-Fragments	Recipient Chromosome (A/C)	Size of the Recipient (A/C) Chromosome (Mb)	Size of the Introgressed B-Fragment (Mb)	Relative Size of the Introgressed Fragment (%)	Putative B-Chromosome Associated*^a^*
IL7	1	C3	67.05	18.73	27.93	—
IL13	1	A9	37.98	4.25	11.18	B6
IL16	1	A9	37.98	4.52	11.90	B6
IL20	1	A3	35.76	3.44	9.62	—
IL27	1	C9	52.92	20.53	38.79	B6/B7
IL39	1	C9	52.92	3.99	7.54	B4/B6
IL43	2	C5	46.89	4.36	9.30	—
—	—	C8	42.97	6.70	15.60	—
IL51	1	C5	46.89	6.87	14.65	—
IL54	1	C2	51.34	42.44	82.66	B7
IL59	4	A9	37.98	3.00	7.90	B6/B7
—	—	C3*^b^*	67.05	20.96	31.26	B6/B7
—	—	C8	42.97	3.11	7.23	B6/B7
IL61	1	C9	52.92	23.65	44.70	B6
IL63	1	A3	35.76	3.55	9.93	—
IL69	3	C2	51.34	12.33	24.01	—
—	—	C5	46.89	4.97	10.60	—
—	—	C6	40.57	4.35	10.72	—
IL71	1	A10	19.66	4.25	21.63	—
IL74	1	C1	43.24	7.20	16.66	—
IL75	1	C3	67.05	18.88	28.16	B7
IL78	1	C8	42.97	3.78	8.81	—

a As per B-genome-specific SSRs.

b Chromosome C3 has two introgressed B-fragments of the collective size of 20.96 Mb.

### SNP genotyping to delineate introgressed segments

A stringent criterion (size ≥3.0 Mb) followed to classify a translocation allowed the detection of 23 substituted segments into 17 ILs. These substituted chromosome segments are enumerated in Table 2 and depicted through the circular genome data visualization software (CIRCOS) (Krzywinski *et al.* 2009) in Figure 3. Seven C-genome chromosomes (C1, C2, C3, C5, C6, C8, and C9) and three A-genome chromosomes (A3, A9, and A10) showed substituted B-chromosome fragments in different ILs. The majority of the ILs (14 of the 17) had only one B-fragment (≥3.0 Mb) introgression; two ILs (IL43, IL69) had two chromosomes showing introgressions while only one IL (IL59) had three different chromosomes with B-fragments. The size of the substituted B-genome fragments varied from 3.0 Mb (A9, IL59) to 42.44 Mb (C2, IL54). Of all the 10 chromosomes showing introgressed B-fragments, only one IL (IL59) was identified to be carrying more than one fragment of ≥3.0 Mb size, on chromosome C3. In terms of proportions, the substituted segments ranged from 7.23% (C8, IL59) to almost 83% (C2, IL54) of the actual size of the recipient A/C chromosomes. Only eight (IL13, IL16, IL27, IL39, IL54, IL59, IL61, and IL75) of the 17 ILs showing the presence of B-genome fragments on the basis of SNP assay were common to SSR analysis. In the remaining nine ILs, the absence of such correspondence may be due to the very stringent criterion followed for marking an introgression on the basis of SSR markers (*i.e.*, amplification of two or more consecutive markers on the same chromosome).

### Variation for siliqua shatter resistance

There was significant variation for siliqua strength in the 81 phenotyped ILs. The range varied from 2.2 to 6.0 mJ (timely sown 2012–2013), from 2.7 to 5.5 mJ (late sown 2012–2013), and from 3.1 to 5.0 mJ (timely sown 2013–2014), respectively, in the three experiments. In comparison, the range estimates for the 15 *B. napus* parents varied from 1.8 to 4.0 mJ, from 1.9 to 3.8 mJ, and from 1.8 to 3.9 mJ, respectively. The shatter energy range for the donor parent *B. carinata* was observed to be between 5.6 and 7.2 mJ for the three test environments. Average shatter energy over the environments for the 69 ILs is depicted as histograms on the CIRCOS (Figure 3). Siliqua strength in some of the ILs, namely, IL7 (5.7 mJ), IL8 (5.6 mJ), IL9 (6.0 mJ), IL12 (5.8 mJ), IL16 (5.5 mJ), IL18 (5.9 mJ), IL22 (5.5 mJ), IL23 (6.0 mJ), IL32 (5.7 mJ), IL41 (6.0 mJ), IL48 (5.8 mJ), IL52 (6.0 mJ), IL53 (6.0 mJ), IL63 (5.5 mJ), IL68 (6.0 mJ), IL72 (5.5 mJ), IL75 (6.0 mJ), and IL76 (5.5 mJ), was almost equal to that of the donor parent *B. carinata*.

## Discussion

Crop *Brassica* diploids are ancient polyploids which evolved from a paleo-genome through genome duplication, rearrangements, and fractionation (Warwick and Black 1991; Lagercrantz 1998; Lysak *et al.* 2005). Of the three *Brassica* genomes, the A- and C- genomes are very closely related while the B-genome is phylogenetically distant. Although Panjabi *et al.* (2008) have demonstrated some degree of homeology between the B- and A-/C-genomes; homeologous pairing between B-genome chromosomes with either of the A- and C-genomes is rare (Mason *et al.* 2010, 2011). This poses a challenge to attempts at transfer of desirable traits from the three B-genome-containing species to *B. napus*. Some studies have nevertheless achieved transfer of desirable traits. Chèvre *et al.* (1997) stably introgressed blackleg resistance encoded by genes located on the B-genome of *B. juncea* into *B. napus*. Plieske *et al.* (1998) found that B-genome genes for resistance to blackleg from *B. nigra*, *B. juncea*, and *B. carinata* were introgressed at the same location on the *B. napus* genome. It was argued that this occurred due to the three species sharing the same copy of the resistance gene with sufficient colinearity of the genomic region containing the resistance gene between the B-genome and *B. napus* to allow recombination to occur. In contrast, Fredua-Agyeman *et al.* (2014) could not achieve B-genome introgression from *B. carinata* into *B. napus*. They found intact or broken B-genome chromosomes inherited together with the *B. napus* chromosomes over several generations. This was broadly confirmatory of an earlier study by Navabi *et al.* (2011) who found no evidence of B-genome chromosome introgression into the A- or C- chromosomes of *B. napus*. They also showed that B-genome chromosomes were inherited as a whole linkage group, with the occasional loss of terminal segments, and several of the B-genome chromosomes were retained across generations. Although Chèvre *et al.* (1991) have shown excellent transmission frequency (50–100%) of B-genome chromosomes in *B. napus*–*B. nigra* disomic chromosome addition lines, low transmission of translocated chromosomes during early stages of the backcross program may be the reasons for failure to introgress B-genome fragments in previous studies. Lack of selection pressure for retention of B-chromosomes during initial backcross generations and small sample size of backcross or selfed progenies may have been the other limiting factors.

A strategy of parallel backcrossing, selfing, and phenotypic selection supplemented with marker assisted selection was followed for developing the ILs. Retention of a higher proportion of the donor B-genome was favored by limiting the cycles of backcrossing to two. This was followed by five cycles of selfing and phenotypic selection for hard to thresh siliquae. A very large population base in each selfing cycle seemed crucial for this scheme. Employing a large set of *B. napus* parents was opted for making BC_2_ crosses to facilitate introgression while importing more genetic diversity from the *B. napus* genome. Preselection in backcross generations based either on genotypic or phenotypic selection was followed which has also been proved successful in other crops (Eshed and Zamir 1994; Eduardo *et al.* 2005).

Despite selection for high pollen fertility in each recombination cycle, pollen grain stainability in the 69 ILs varied from 60 to 97.5%. High pollen fertility is normally considered as a reflection of stable meiosis, which was confirmed from meiotic analysis wherein 19 normal bivalents with regular anaphase chromosome separation were observed in all the ILs. It is likely that selection pressure for improved pollen grain fertility helped to stabilize translocations as translocation homozygotes. GISH confirmed the presence of large (≥3.0 Mb) B-genome fragments for up to four *B. napus* chromosomes. Amplification with B-genome SSR markers suggested fragment substitutions in 39 ILs showing introgressed fragments from only four (B3, B4, B6, and B7) of the eight B-genome chromosomes. There appeared to be no retention of intact B-genome chromosomes. In a few cases, the substituted B-genome fragments were from terminal regions of the B-genome chromosomes. B-genome markers of two B-chromosomes from interstitial and terminal regions were present in IL59. These emphasized the possibility of large and multiple translocations resulting from chromosome breakage and reunions. Their presence also indicates that chromatin substitutions must have occurred in BC_1_ generation, as different recipient *B. napus* parents were used for producing the BC_2_. ILs carrying fragments from chromosome B3 may be of special interest for canola breeders due to reported association of *B. carinata* chromosomes B3 and B8 with cotyledon stage resistance against blackleg (Fredua-Agyeman *et al.* 2014).

B-genome segment substitution was detected in 7 of the 9 C-genome chromosomes (C1, C2, C3, C5, C6, C8, and C9) and in 3 of the 10 A-genome chromosomes (A3, A9, and A10). The size of the substituted B-genome fragments varied from 3.00 Mb (A9, IL59) to 42.44 Mb (C2, IL54). This translates into 7.23% to almost 83% of the recipient A/C chromosomes, depending on the size of the recipient chromosome. Due to the stringent criterion followed to classify a translocation (minimum 3.0 Mb size of substituted fragment), SNP genotyping provided a conservative estimate of B-genome presence. The introgressed fragments were scattered throughout the recipient chromosome. This may have resulted from preferential chromosome recombination events between areas of high homeology, plus selection pressure for the target trait during development of ILs. Several mapping studies in the past have demonstrated homologous regions that the B-genome chromosomes shared with the A- and C-genomes (Lagercrantz and Lydiate 1996; Panjabi *et al.* 2008). Comparative mapping of different Brassicaceae lineages has illustrated the presence of conserved genome blocks in *B. napus* (Parkin *et al.* 2005; Schranz *et al.* 2006). Molecular characterization of *B. napus*, resynthesized through *B. juncea* × *B. carinata* hybridizations, had also revealed introgressed B-genome segments (Chatterjee *et al.* 2016) from all the B-chromosomes but more so from B6. More recently, Gupta *et al.* (2016) have reported a sizeable number of C-genome chromosome substitution lines in the progenies of derived *B. juncea*, suggesting that the substituting C-genome chromosomes were likely to have replaced the B-genome chromosomes.

Many ILs revealed a very high rupture energy requirement, which was almost as good (6.0 mJ) as that recorded for *B. carinata* (7.2 mJ), the donor parent. Special mention may be made of IL9, IL23, IL41, IL52, IL53, IL63, and IL68. Transferability of introgressed shatter resistance was also confirmed in the progenies of crosses involving ILs and several genotypes of euploid *B. napus*.

Introgression lines constitute permanent diversity conduits for crop improvement. Apart from their use as a plant breeding resource, these have been utilized for identifying genes (Eshed and Zamir 1996), analyzing pleiotropic effects, differentiating between pseudo overdominance and true-dominance (Yamamoto *et al.* 1998), and map-based QTL cloning. Phenotypic variation and presence of a significant proportion of the donor genome in the ILs is critical for their optimal utilization. Frey *et al.* (1983) were the first to recognize the productivity-increasing potential of alien genes in oats. This was followed by the synergistic association of QTL mapping and alien gene introgression conceived by Tanksley *et al.* (1996), who also suggested the advanced backcross QTL strategy. The ILs reported in our study can be used to construct a new platform for genetic and functional genomics analysis in *B. napus*. These contain substituted B-genome segments on the C-/A-genome chromosomes and may carry QTL for key productivity or defense-related traits. However, linkage drag, distal location of the translocated segment, interaction with recipient chromosome(s), transmission frequency of translocated chromosome, and pleiotropy may limit their utility. It may still be possible to develop fine ILs carrying a single and small introgressed segment for future use in advanced backcrossing and marker-assisted programs. Developing secondary F_2_ or DH populations through hybridization between ILs and recipient parents may be another option to fine map introgressed QTL (Tian *et al.* 2006). The ILs show significant differences for many yield-related traits, especially siliqua shatter resistance, and will be used in future to map the genes/QTL controlling productivity and siliqua shattering. Further investigations may involve complete genome or transcriptome sequencing to rapidly identify possible candidate genes in the tagged QTL regions.

## Supplementary Material

Supplemental material is available online at www.g3journal.org/lookup/suppl/doi:10.1534/g3.116.036442/-/DC1.
