# Supplementary figures and images for: Cytogenetic and Molecular Characterization of B-Genome Introgression Lines of Brassica napus L
Source: G3 (Bethesda). 2016 Nov 7;7(1):77–86. doi: 10.1534/g3.116.036442 (PMC5217125; doi:10.1534/g3.116.036442)

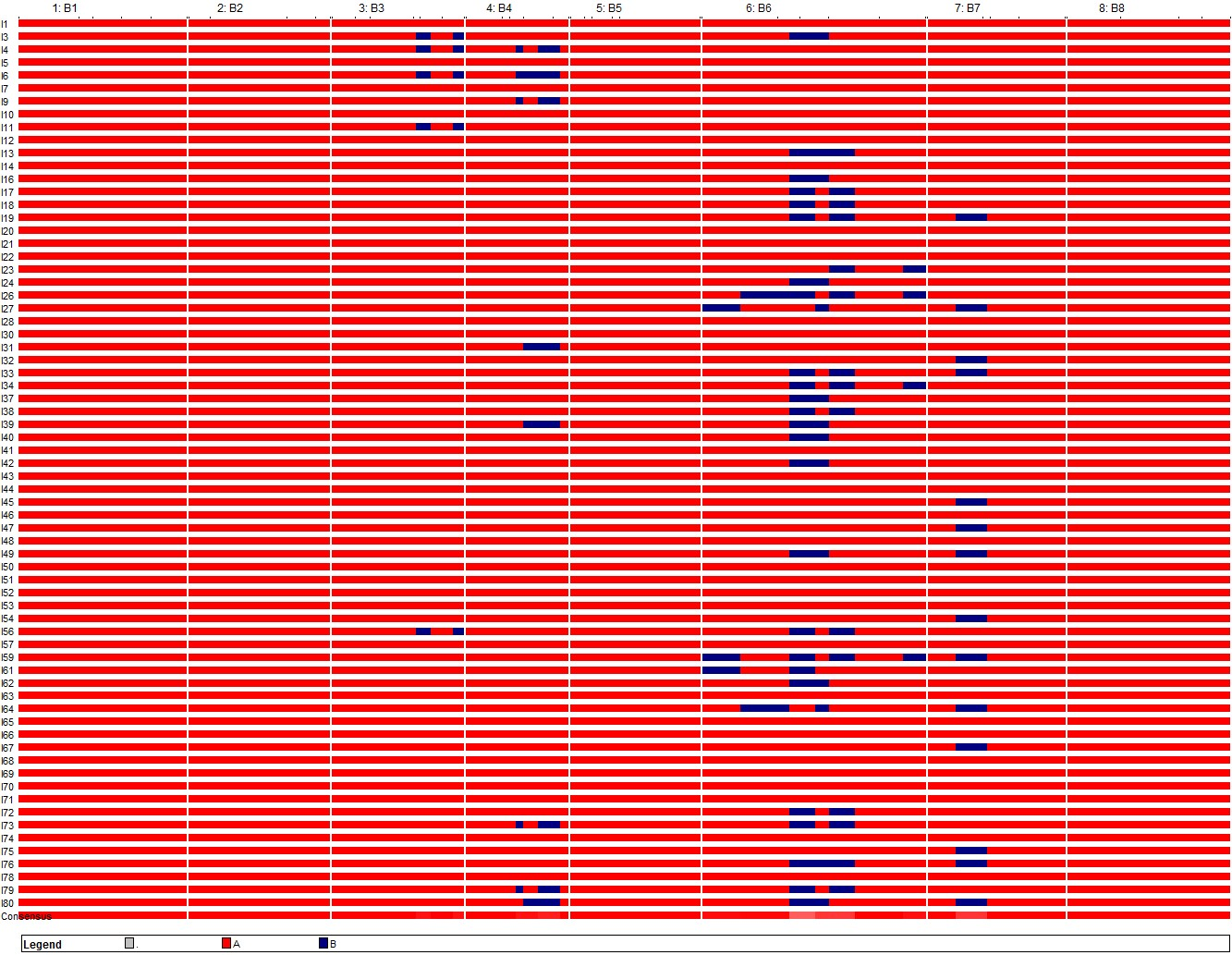

Supplement: Supplementary file 1 [file 77file001.tif]

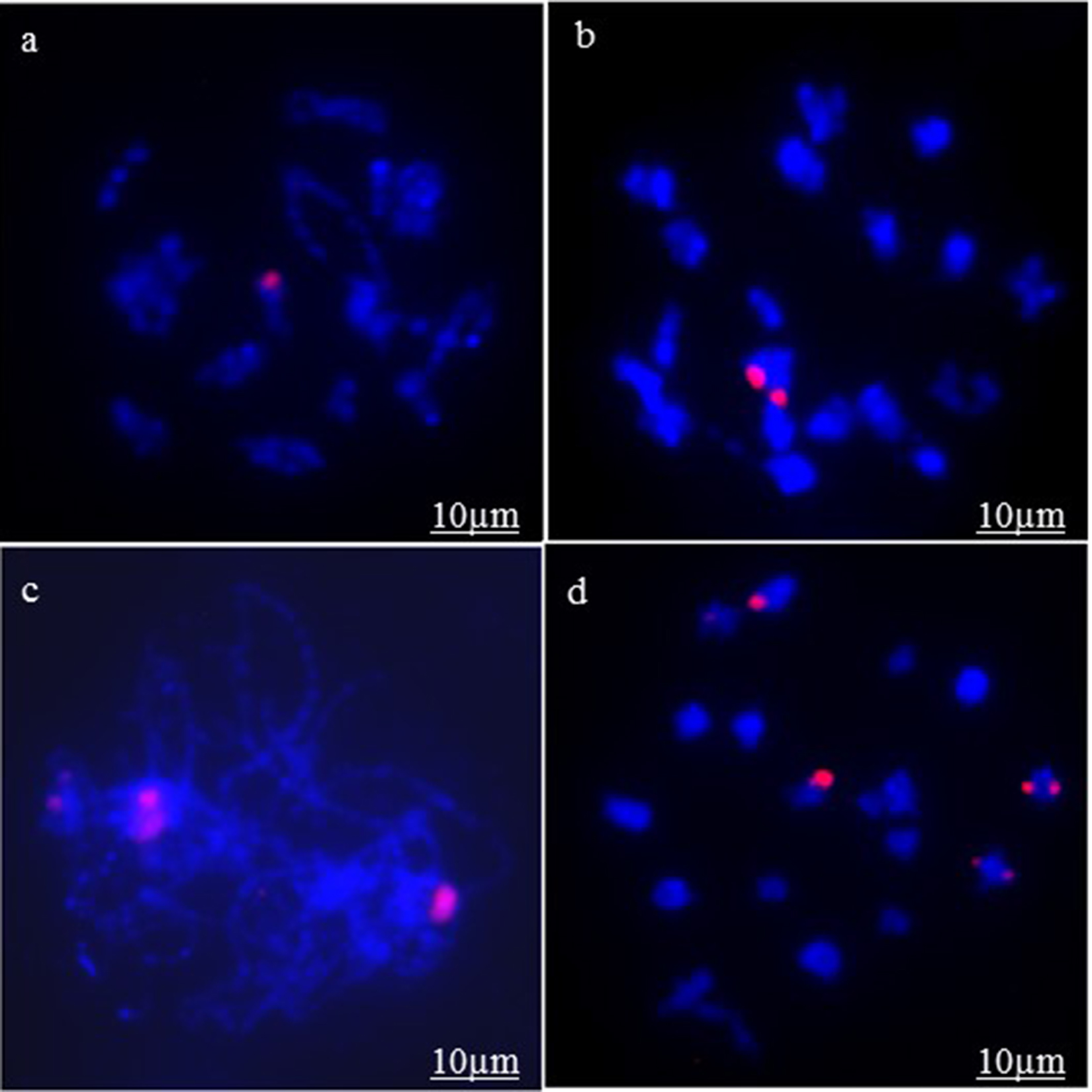

Supplement: Supplementary file 2 [file 77file002.tif]

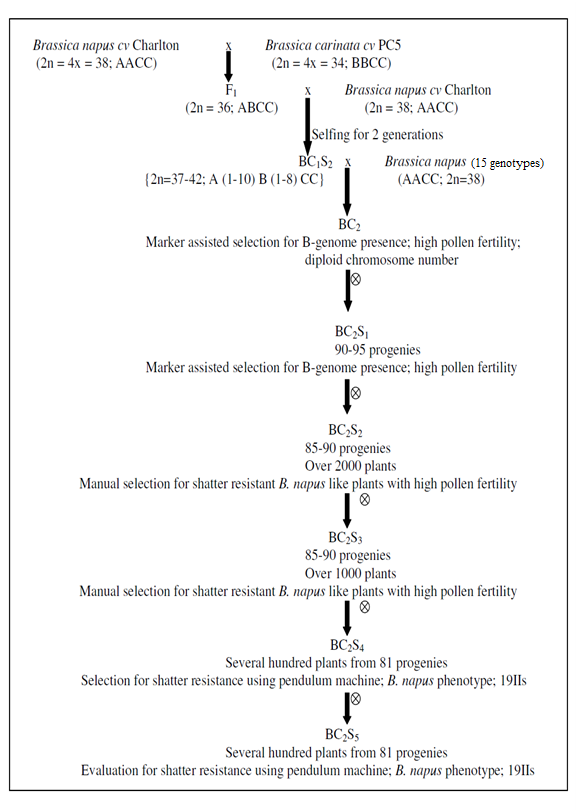

Supplement: Supplementary file 3 [file 77file004.tif]

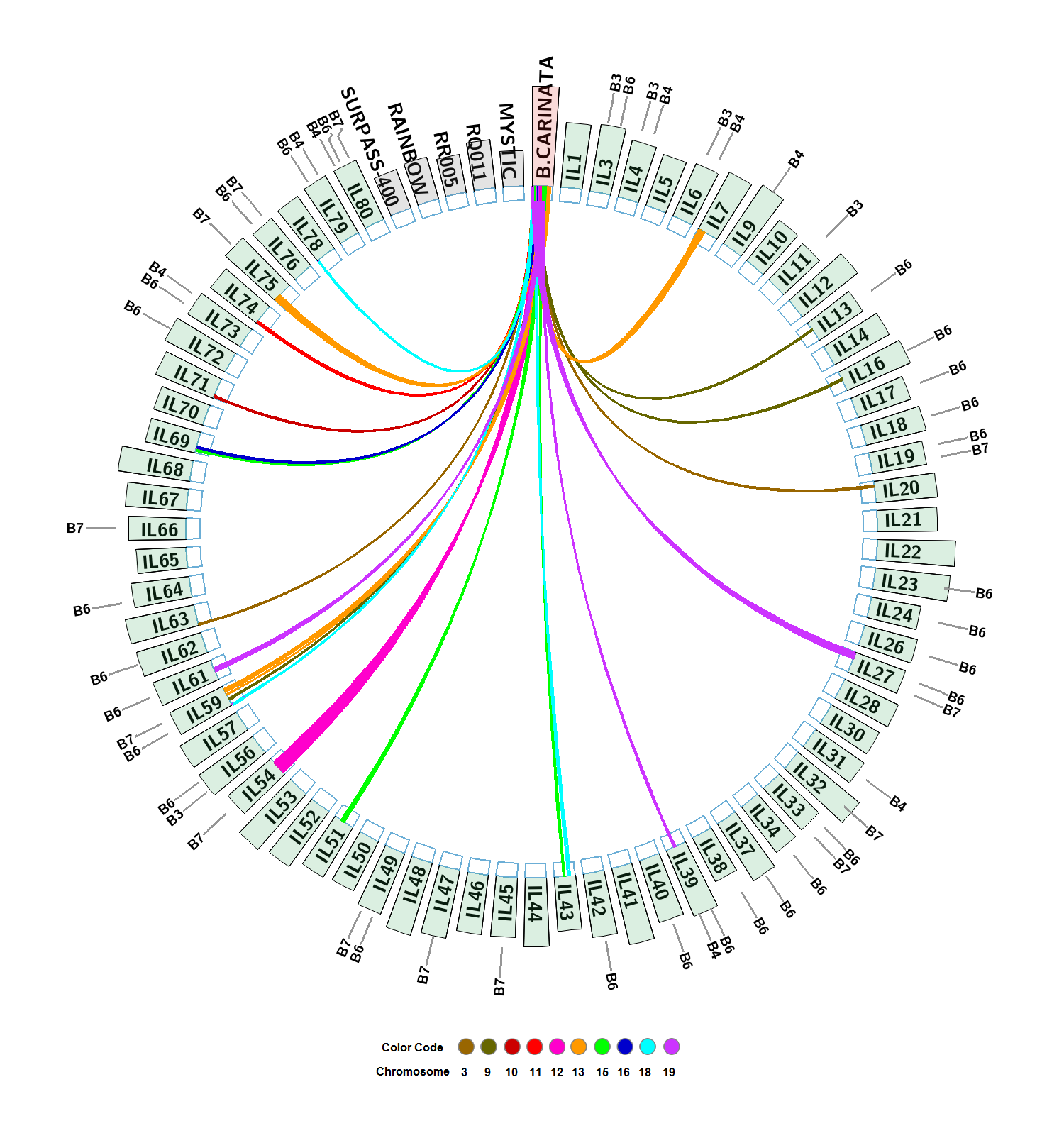

Supplement: Supplementary file 4 [file 77file005.tif]
